# Exploratory Genomic Characterisation of the Resistome, Virulome and Molecular Epidemiology of Multidrug-Resistant *Klebsiella pneumoniae* Clinical Isolates from Four Peruvian Regions

**DOI:** 10.3390/microorganisms14071573

**Published:** 2026-07-19

**Authors:** Karen Quispe Oré, Alberto Salazar-Granara, Mario Cueva Távara, Janet Huancachoque Molina, Diego Segura-Loayza, Daisy Obispo Achallma, Guillermo Liendo Aguirre, Antonio Burgos Espejo, Alexander Briones Alejos, Sheyber Lifonzo Mucha, Teresa Alarcón-Castillo, Pool Marcos-Carbajal

**Affiliations:** 1 Universidad Nacional Mayor de San Marcos, Facultad de Ciencias Biológicas, Lima 15081, Peru; karen.quispe1@unmsm.edu.pe (K.Q.O.);; 2 Universidad de San Martin de Porres, Centro de Investigación de Medicina Tradicional y Farmacología, Facultad de Medicina Humana, Lima 15001, Peru; asalazarg@usmp.pe (A.S.-G.); janet_hm@hotmail.com (J.H.M.); diegoseguraloayza@gmail.com (D.S.-L.); 3 Universidad de San Martin de Porres, Centro de Investigación en Genética y Biología Molecular, Facultad de Medicina Humana, Lima 15001, Peru; dobispoa@usmp.pe; 4 Hospital Regional de Moquegua, Moquegua 18001, Peru; blgoguillermo2023@gmail.com; 5 Hospital Regional de Pucallpa, Ucayali 25001, Peru; segundo67lab@hotmail.com; 6 Hospital Regional de Loreto, Loreto 16004, Peru; alexander.brionesalejos@gmail.com; 7 Hospital de Huaycan, Lima 16007, Peru; sheybrt@gmail.com; 8 Universidad Nacional Intercultural de la Amazonía Facultad de Ingeniería y Ciencias Ambientales, Departamento Académico de Ciencias Básicas, Campus Puerto Callao, Ucayali 25006, Peru; talarconc@unia.edu.pe

**Keywords:** virulence factors, multidrug resistance genes, *Klebsiella pneumoniae*, WGS

## Abstract

Multidrug-resistant (MDR) *Klebsiella pneumoniae* is a critical cause of nosocomial infections associated with high mortality. Of particular concern is the genomic convergence of multidrug resistance and hypervirulence (MDR-hvKP), which represents a severe public health threat. This study characterised the resistome, virulome, and population structure of seven clinical MDR *K. pneumoniae* isolates collected between 2024 and 2025 across four Peruvian regions (Moquegua, Ucayali, Loreto, and Lima) using whole-genome sequencing (WGS). Genomic analysis identified six distinct sequence types, with the high-risk clone ST307 being the most prevalent (2/7 isolates), consistently associated with the KL102 capsular locus. Remarkably, one isolate from Loreto (oph_54) exhibited a convergent MDR-hvKP genomic profile (ST218, KL57, virulence score 4), carrying yersiniabactin, salmochelin (*iro*), and aerobactin (*iuc*) loci alongside multiple resistance determinants. The isolates presented a robust resistome dominated by the extended-spectrum beta-lactamase gene *bla*_CTX-M-15_ (5/7) and *bla*_OXA-1_, coupled with *aac(6′)-Ib-cr* and *fosA6* genes. Plasmid analysis revealed a predominance of IncFIB(K) replicons. Although exploratory, these findings demonstrate the regional presence of high-risk ST307 lineages and the emergence of convergent MDR-hvKP genomic profiles within the analysed Peruvian hospitals. Immediate and continuous genomic surveillance is urgently required to monitor the potential spread of these highly dangerous, MDR bacterial lineages within these healthcare environments.

## 1. Introduction

*Klebsiella pneumoniae* is a Gram-negative bacterium that belongs to the *Enterobacteriaceae* family and is a major cause of community- and hospital-acquired infections, including pneumonia, bloodstream infections and urinary tract infections [1]. The widespread and often inappropriate use of antibiotics has led to the rapid emergence of multidrug-resistant (MDR) *K. pneumoniae*, severely limiting treatment options. In recent years, the clinical management of these infections has shifted dramatically; traditional regimens relying on highly toxic polymyxins (renal damage) have been superseded by novel β-lactam/β-lactamase inhibitor combinations, such as ceftazidime-avibactam, or complex multi-drug combination therapies. However, recent global evidence underscores that the clinical success of these contemporary treatments is continuously undermined by regional variations in molecular resistance mechanisms [2,3]. At the same time, certain lineages have evolved into hypervirulent pathotypes, which further complicates clinical management, particularly in low- and middle-income countries [4]. Globally, the prevalence of nosocomial MDR *K. pneumoniae* has been estimated at 32.8%, while in South America it reaches alarmingly high levels of up to 72.4% [5]. Infections caused by MDR strains are associated with mortality rates of almost 50% [6]. The most frequently reported risk factors are haematological malignancies, prolonged hospitalisation in intensive care units (ICUs) and prior exposure to broad-spectrum antibiotics such as β-lactams, fluoroquinolones, polymyxins, sulfonamides and tetracyclines [7]. In Peru, surveillance studies have reported a prevalence exceeding 70% for MDR *K. pneumoniae* at the National Hospital Ramiro Prialé Prialé, located at high altitude in the central Andes, between 2012 and 2018, highlighting the scale of the issue in the country, although these data come from a single tertiary care hospital, they highlight the high prevalence and heterogeneity of MDR *K. pneumoniae* in Peru [8]. Beyond resistance gene carriage, clinical MDR *K. pneumoniae* isolates from infected wounds have demonstrated complex resistance profiles even in resource-limited settings, underscoring the widespread distribution of MDR lineages across diverse clinical sources, including wounds and urinary tract infections [9,10]. Furthermore, the capacity of *K. pneumoniae* to form biofilms, a trait closely linked to antibiotic treatment failure, has been recognised as an additional dimension of pathogenicity that amplifies resistance phenotypes in hospital environments [11,12].

*K. pneumoniae* can be broadly classified into two main pathotypes: classical *K. pneumoniae* (cKP) and hypervirulent *K. pneumoniae* (hvKP) [13]. The accessory genome plays a critical role in distinguishing between these pathotypes, as well as in defining their antimicrobial resistance and virulence profiles [14]. Key virulence factors include the polysaccharide capsule, lipopolysaccharide (LPS), fimbriae, siderophore systems, outer membrane proteins, secretion systems, colibactin, and the AcrAB efflux pump, to name a few [15,16]. Of particular concern is the convergence of hypervirulence and carbapenem resistance, which is often mediated by hybrid plasmids carrying both virulence and resistance determinants. This leads to the emergence of highly transmissible ‘superbug’ clones, such as carbapenem-resistant hypervirulent *K. pneumoniae* (CR-hvKP), which have been widely reported in Asia [17].

Whole-genome sequencing (WGS) is a powerful tool for comprehensively characterising MDR *K. pneumoniae*. It enables the identification of a wide range of resistance factors, including β-lactamases, efflux systems, porin alterations and mutations at target sites [18,19]. Furthermore, WGS enables the study of mobile genetic elements, such as plasmids, bacteriophages and transposons, that facilitate the horizontal transfer of resistance and virulence genes via conjugation and transduction. This accelerates bacterial adaptation and dissemination [20].

However, in Peru, most reports on *K. pneumoniae* epidemiology are limited to phenotypic antimicrobial susceptibility testing. Despite the country’s high burden of antimicrobial resistance and associated mortality, relatively little genomic data is available [21]. The emergence of carbapenemase-producing *K. pneumoniae* (CRKP) poses an increasing threat to public health. The first cases of systemic infections caused by *K. pneumoniae* producing OXA-48 were reported in Peru in 2021 [22].

Subsequently, Cuicapuza et al. reported the presence of *K. pneumoniae* producing KPC ST11 in a Peruvian hospital, highlighting the presence of a high-risk international clone [23]. In 2025, *K. pneumoniae* isolates carrying the *bla*_NDM_ and *bla*_KPC_ genes, along with high-risk clones (ST45 and ST348) that are rarely seen elsewhere in South America, were found at a hospital in Lima [24].

The present study aims to characterise the genomes of clinical MDR *K. pneumoniae* isolates from four Peruvian hospitals using whole-genome sequencing (WGS) to identify resistance genes, virulence factors, plasmids, and prevalent sequence types (STs).

## 2. Materials and Methods

### 2.1. Klebsiella pneumoniae MDR Isolates

Seven *K. pneumoniae* MDR isolates were analysed. These were obtained from four Peruvian hospitals: Huaycán Hospital (Lima Region, *n* = 1); Loreto Regional Hospital (Loreto Region, *n* = 2); Moquegua Regional Hospital (Moquegua Region, *n* = 1); and Pucallpa Regional Hospital (Ucayali Region, *n* = 3). The isolates were derived from two clinical sources, urine and blood (Table 1). This was an exploratory pilot study focused strictly on multicentre, highly resistant clinical isolates recovered during a specific surveillance window (2024–2025). The strict phenotypic selection criteria, combined with logistical and resource constraints associated with whole-genome sequencing (WGS) in remote geographic regions of Peru, determined the final sample size. The MDR isolates were identified using the VITEK 2 Compact system (bioMérieux, Marcy-l’Étoile, France) with MicroScan Neg Panel Type 41 (Beckman Coulter, Brea, CA, USA) or disk diffusion tests. Multidrug resistance (MDR) was defined as non-susceptibility to at least one agent in three or more antimicrobial classes, according to the criteria proposed by Magiorakos and coworkers, using CLSI M100 guideline for susceptibility interpretation [25,26]. Detailed phenotypic AST profiles, including methods, antimicrobial panels, and raw values, are provided in Supplementary Table S1. The samples were transported in tubes containing TSA medium under a cold chain (2–8 °C) to the Centre for Research in Traditional Medicine and Pharmacology at the Faculty of Human Medicine, Universidad de San Martín de Porres. The samples were then cultured on the following media: MacConkey, Chromagar^®^ mSuperCarba and ESBL (CHROMagar, Paris, France), to identify *K. pneumoniae* and evaluate resistance.

### 2.2. DNA Extraction

Genomic DNA from the isolated strains was extracted using the Wizard^®^ Genomic DNA Purification Kit (Promega, Madison, WI, USA) following the manufacturer’s protocol. The DNA quantity was determined using Qubit 2.0 (Thermo Fisher Scientific, Waltham, MA, USA), and its quality was evaluated using NanoDrop 2000 (Thermo Fisher Scientific).

### 2.3. Whole-Genome Sequencing (WGS)

Library preparation for sequencing was performed using the Native Barcoding Kit 24 V14 (SQK-NBD114.24, Oxford Nanopore Technologies, Oxford, UK), which allows for native DNA sequencing without prior amplification. A unique barcode adapter was ligated to the DNA fragments, and the library was purified before being stored at 4 °C until use. Sequencing was performed using the MinION Mk1B platform (Oxford Nanopore Technologies) equipped with a R10.4.1 MinION Flow Cell (FLO-MIN114). The flow cell was loaded with the DNA library, and sequencing was carried out using the Native Barcoding Kit 24 V14.

### 2.4. Bioinformatic Analysis

Basecalling was performed using the integrated Dorado v7.8.3 (https://github.com/nanoporetech/dorado, accessed on 22 September 2025) basecaller within the MinKNOW software suite v25.03.9, Bream v8.4.4 and MinKNOW Core v6.4.9 with configuration v6.4.11, utilising the high-accuracy model (HAC, 400 bps) with a minimum quality score (*Q*-score) cutoff of 9. To ensure the complete removal of any residual sequencing adapters, Porechop v0.2.4 (https://github.com/rrwick/Porechop, accessed on 23 September 2025) was applied to the basecalled reads. Data were then refined using NanoFilt v2.8.0 (https://github.com/wdecoster/nanofilt, accessed on 23 September 2025) for quality and length filtering, with final quality control assessment provided by Nanoplot v1.46.2 (https://github.com/wdecoster/NanoPlot, accessed on 23 September 2025) [27]. Genome assembly was done using Flye v2.8.1 (https://github.com/mikolmogorov/Flye, accessed on 23 September 2025) [28]. Assembly quality was improved using Medaka v1.6.1 (https://github.com/nanoporetech/medaka, accessed on 24 September 2025) with the r1041_e82_hac_g415 model which precisely matches the R10.4.1 flow cell chemistry (FLO-MIN114) utilised during the sequencing runs. Assembly quality was assessed using QUAST v5.0.2 (https://github.com/ablab/quast, accessed on 24 September 2025). Through the Galaxy Europe/Australia platform, genomic completeness and contamination were estimated using CheckM2 v1.0.1 (https://github.com/chklovski/CheckM2, accessed on 20 April 2026) [29,30]; only genomes with 100% completeness and <3% contamination were retained, and species identification was performed using Kraken2 v2.17.1 (https://github.com/DerrickWood/kraken2, accessed on 20 April 2026). Similarly, the Average Nucleotide Identity (ANI) was calculated using FastANI v1.34 (https://github.com/ParBLiSS/FastANI, accessed on 20 April 2026) via Galaxy to evaluate genomic relatedness. Genomic characterisation of *K. pneumoniae* isolates, including sequence typing, was carried out using Kleborate v2.3.2 [31]. Antimicrobial resistance and virulence genes were further screened using ABRicate v1.2.0 (https://github.com/tseemann/ABRICATE, accessed on 29 September 2025) with the ResFinder and VFDB databases, respectively. Plasmid reconstruction and typing were performed using MOB-suite v3.1.9 (https://github.com/phac-nml/mob-suite, accessed on 29 September 2025) and PlasmidFinder (https://github.com/genomicepidemiology/plasmidfinder, accessed on 29 September 2025), Capsular loci and serotypes were determined using Kaptive v3.2.1. (https://github.com/klebgenomics/Kaptive, accessed on 29 September 2025). MLST was additionally confirmed using MLST v2.23 (https://github.com/tseemann/mlst, accessed on 29 September 2025). Genome annotation was performed using the NCBI Prokaryotic Genome Annotation Pipeline (PGAP) [32].

### 2.5. Phylogenetic Analysis

The phylogenetic relationships among the isolates were evaluated based on core genome SNP alignments. Initially, draft genome assemblies were mapped against the reference genome *K. pneumoniae* HS11286 using Snippy v4.6.0. (https://github.com/tseemann/snippy, accessed on 21 April 2026) with default parameters (minimum mapping quality of 60, minimum depth of 10x, and a variant allele frequency threshold of 0.90). Individual alignments were consolidated into a core-genome matrix using snippy-core. To eliminate potential systematic base-calling artefacts, homopolymer errors inherent to long reads, and genuine recombination events, the core alignment was filtered using Gubbins v3.1.6. Phylogenetic tree reconstruction was performed using IQ-TREE v3.0.1 from the filtered polymorphic site matrix. The GTR + ASC + G substitution model was explicitly applied to mathematically account for the exclusive use of polymorphic data and avoid branch-length overestimation. Branch support was assessed using 1000 ultrafast bootstrap replicates (-bb 1000). The final tree topology was visualised using iTOL v7.0 and rooted via the midpoint strategy [33,34].

### 2.6. Statistical Analysis

Due to the small sample size (*n* = 7), analyses were descriptive and exploratory. The similarity matrix generated from the ANI analysis was visualised through a heatmap using the pheatmap package in R v4.4.2. (GitHub—https://github.com/raivokolde/pheatmap, accessed on 25 April 2026). To group the isolates based on their genomic identity, a hierarchical clustering analysis was performed using the complete linkage method. Differences in genome metrics and gene counts were summarised using descriptive statistics. To evaluate intra-isolate resistome heterogeneity, Simpson’s Index of Diversity (1 − *D*) was calculated for each clinical isolates using the finite community framework: 1−D= 1−[∑n(n−1)/N(N−1)], where n is the number of resistance genes within a specific antimicrobial class (Aminoglycosides, Beta-lactams, Quinolones, Sulfonamides, Trimethoprim, Fosfomycin, and Tetracyclines) and N is the total AMR gene count for that individual isolate [35].

### 2.7. Ethical Considerations

The research protocol was approved by the Institutional Ethics Committee of the Faculty of Human Medicine, Universidad de San Martín de Porres, with reference number No. 653-2024-CIEI-FMH-USMP.

## 3. Results

### 3.1. Plasmids

A total of 11 distinct plasmid replicon types were detected, with all isolates harbouring multiple plasmids (ranging from three to six replicons per genome). The reconstruction revealed a highly dynamic extrachromosomal architecture dominated by large conjugative megaplasmids (100.3–419.7 kb) belonging to the IncFIB, IncFII, and IncHI1B families (Table 2). Genomically, the IncFIB(K)_1_Kpn3 replicon was the most prevalent, identified in 6/7 isolates, followed by ColRNAI_1, IncFIB(Mar), and IncHI1B_1_pNDM-MAR (each in 4/7 isolates). Notably, IncHI1B_1_pNDM-MAR was consistently co-localised on the same physical scaffolds with IncFIB(Mar)_1_pNDM-Mar, confirming they form massive multi-replicon MDR platforms.

These critical MDR hubs were successfully mapped onto individual physical structures. Specifically, the conjugative plasmid plasmid_AA405 was identified as a major vehicle circulating in both oph_35 (419.7 kb) and oph_43_r (264.8 kb), harbouring dense arrays of up to 14 AMR genes, including *bla*_CTX-M-15_, aminoglycoside-modifying enzymes (*aac*, *aph*), and fluoroquinolone determinants. Similarly, the 228.9 kb conjugative plasmid_AA274 in oph_14 concentrated 12 AMR genes, notably carrying *bla*_CTX-M-27_ and *bla*_DHA-1_.

Beyond these dominant megaplasmids, a diverse array of accessory elements was observed, including IncFII variants, IncR, IncX1, IncN, and several small Col-type units (Col440I, ColE10). The frequent coexistence and complex architecture of these mobile elements underscore their role as potential basal platforms ready for further resistance gene acquisition within the clinical ecosystem.

### 3.2. Phylogenetic Tree, Typing, and Resistance Profiles of the Isolates

**Figure 1:**
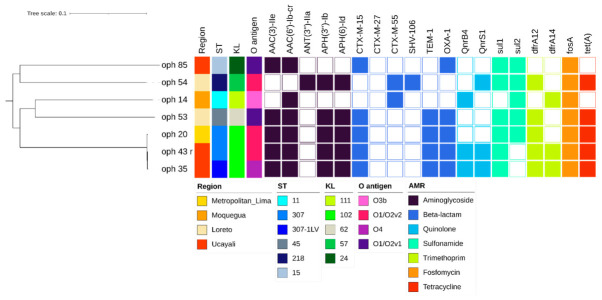
Core-genome SNP-based phylogenetic tree and genomic characterisation of the seven *K. pneumoniae* isolates. The dendrogram (left) illustrates the genetic relatedness among isolates. Maximum-likelihood phylogenetic tree was reconstructed based on core-genome SNP alignments using Snippy and IQ-TREE. The scale bar indicates 0.1 substitutions per nucleotide site. The heatmap (right) displays the distribution of sequence types (ST), capsular loci (KL), and O-antigen types, followed by the AMR gene profiles detected via WGS. Colored boxes indicate the presence of a specific determinant, while white boxes indicate its absence.

The seven clinical MDR *K. pneumoniae* isolates were analysed by whole-genome sequencing to determine sequence types (ST), capsular loci (KL), and lipopolysaccharide (O antigen) (Figure 1). Six distinct ST were identified, including the high-risk clones ST307, ST15, and ST11, with ST307 being the most prevalent (*n* = 2), along with a closely related variant (ST307-1LV, *n* = 1).

A high diversity of capsular types was observed, with five different KL types detected. Notably, ST307 and its single-locus variant ST307-1LV were consistently associated with KL102 but exhibited variation in O-antigens, including O1/O2v2 and O4. In contrast, ST11 was associated with KL111 and O3b. Other STs showed distinct combinations, such as ST45 with KL62 and O1/O2v1, ST218 with KL57 and O1/O2v2, and ST15 with KL24 and O1/O2v1. Overall, the distribution of ST–KL–O combinations highlights the genomic diversity of the isolates.

A wide range of resistance determinants was detected, spanning multiple antibiotic classes, including aminoglycosides, β-lactams, fosfomycin, macrolides, quinolones, sulfonamides, tetracyclines, and trimethoprim. Among β-lactam resistance genes, the extended-spectrum β-lactamase (ESBL) genes of the *bla*_CTX-M_ family were the most prevalent, with *bla*_CTX-M-15_ present in (5/7 isolates), while *bla*_CTX-M-27_ and *bla*_CTX-M-55_ were each detected in (1/7 isolates). Other β-lactamases identified included *bla*_OXA-1_ (5/7 isolates), *bla*_TEM-1_ (4/7 isolates), and the AmpC β-lactamase *bla*_DHA-1_ (1/7 isolates). Aminoglycoside resistance genes included *aac(6′)-Ib-cr* (6/7 isolates), *aac(3)-IIe* (5/7 isolates), *aph(3″)-Ib* (5/7 isolates) and *aph(6)-Ib* (5/7 isolates). No carbapenemase genes were identified. All isolates harboured the *fosA* family genes, specifically the *fosA6* variant, associated with fosfomycin resistance. Quinolone resistance determinants included the plasmid-mediated genes *qnrS1* (2/7 isolates), *qnrB4* (2/7 isolates), and *qnrB17* (1/7 isolates). Sulfonamide resistance genes included *sul1* (4/7 isolates) and *sul2* (6/7 isolates). Tetracycline resistance genes included *tet*(A) (6/7 isolates) and *tet*(D) (1/7 isolates), while trimethoprim resistance was mediated by *dfrA14* (5/7 isolates), *dfrA12* (2/7 isolates), and *dfrA27* (1/7 isolates).

### 3.3. Virulence Profiles

**Figure 2:**
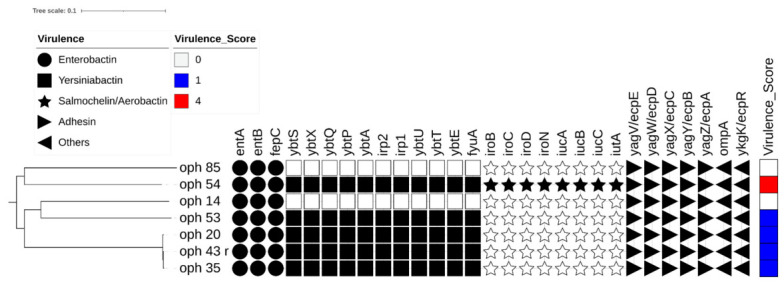
Virulome profile and virulence scores of *K. pneumoniae* isolates. The phylogenetic tree is annotated with the presence (filled symbols) or absence (empty symbols) of key virulence determinants, including siderophores (enterobactin, yersiniabactin, salmochelin, and aerobactin) and adhesins. The heatstrip on the right indicates the Kleborate virulence score: 0 (none/basal), 1 (yersiniabactin), and 4 (yersiniabactin plus aerobactin and/or salmochelin). Isolate oph_54 represents the convergent MDR-hvKP genomic profile.

The seven clinical multidrug-resistant (MDR) *K. pneumoniae* isolates were analysed by WGS to identify virulence genes (Figure 2). All isolates harboured the adhesins *yagV/ecpE*, *yagW/ecpD*, *yagX/ecpC*, *yagY/ecpB*, *and yagZ/ecpA*. More than half (5/7 isolates) of the isolates presented the yersiniabactin (*ybt*) gene loci. Other important virulence genes detected included the *iutA* gene (ferric aerobactin receptor), found in (1/7 isolates) of the isolates. One isolate from the Loreto region (oph_54) was classified as possessing an MDR-hvKP (multidrug-resistant hypervirulent *K. pneumoniae*) genomic profile according to the Kleborate scheme. This isolate belonged to sequence type ST218 with capsular locus KL57. The presence of virulence and resistance genes in this isolate reflects the convergence of multidrug resistance and hypervirulence-associated genetic markers.

### 3.4. Genomic Similarity and Resistome Diversity

**Figure 3:**
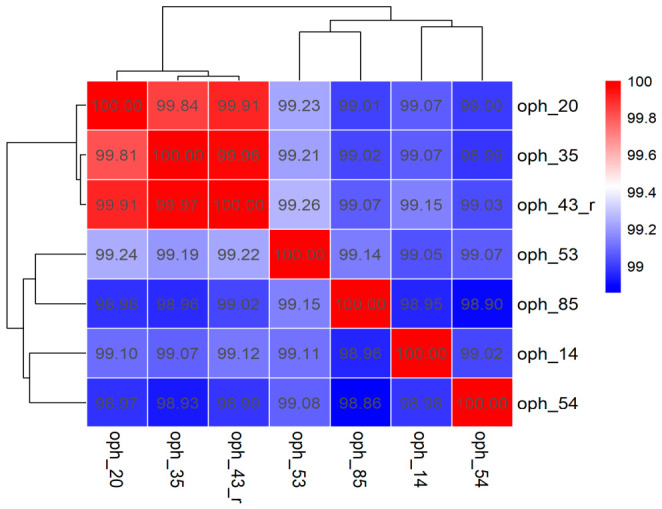
Heatmap of Average Nucleotide Identity (ANI) values among the seven *K. pneumoniae* clinical isolates. The colour scale represents the percentage of genomic identity, ranging from blue (lower identity) to red (higher identity). Hierarchical clustering indicates the genomic relatedness between isolates, highlighting the high similarity within the ST307 cluster.

The average nucleotide identity (ANI) was calculated to assess genomic similarity among the isolates (Figure 3). Pairwise ANI values were computed using FastANI v1.34 with default parameters, comparing all genomes against each other. The heatmap illustrates the high genomic conservation (ANI > 99%). Red blocks indicate nearly identical genomic content, confirming the clonal nature of specific isolates in ST307 lineage found in Lima and Ucayali, while more divergent colours highlight the genetic heterogeneity of the ST11, ST15, and ST218 isolates. Assessment of intra-isolate resistome diversity revealed distinct gene distribution patterns across the collection (Table 3). The Simpson’s Index of Diversity (1 − *D*) ranged from 0.848 to 1.000, indicating a high overall heterogeneity of resistance mechanisms. Notably, isolate oph_14 (ST11) exhibited the maximum possible diversity (1 − *D* = 1.000), reflecting a uniform distribution of single genes across five antimicrobial classes.

## 4. Discussion

Genomic analysis revealed a high diversity of sequence types (STs) among the few isolates examined, identifying six STs: ST11, ST307, ST307-1LV, ST45, ST218, and ST15. However, a cluster of ST307 associated with the KL102 capsular type and O1/O2v2 antigen was observed as the most frequent within this small sample. This lineage was found in isolates from Lima and Ucayali, suggesting a potential clonal relationship between the ST307 isolates in these regions supported by the high ANI values observed. Phylogenetic analysis revealed low genetic divergence between these specific isolates, indicating close relationships between oph_20, oph_35, and oph_43_r. Other more divergent isolates, such as ST11, ST15, and ST218, were also identified, reflecting genetic diversity and the absence of direct epidemiological relationships among the different collection sites.

In this context, the identification of the ST307 clone in multiple isolates in the present study is consistent with recent reports describing the global expansion of this lineage and its multiple introductions into our region [36]. This clone has been associated worldwide with hospital outbreaks and the spread of extended-spectrum beta-lactamases, particularly CTX-M-15.

Moreover, the presence of the ST11 clone in oph_14 from Moquegua is of clinical interest, as this lineage belongs to the CC258 clonal complex, one of the most important groups associated with resistance worldwide. Although the prevalence of this clone varies by region, its detection in Latin American hospital settings has been increasingly reported over the past decade [37].

Our findings regarding the presence of the high-risk ST307 and ST11 clone in Peru are consistent with Cuicapuza et al. (2024) [23] and Krapp et al. (2025) [24], who reported the presence of these lineages in hospital settings in Lima.

Plasmid analysis revealed a possible horizontal transfer of resistance determinants via plasmids. IncF plasmids, known to be typical of high-risk clinical clones, were frequent among the study isolates [38]. The high prevalence of the IncFIB(K) plasmid suggests its stability and adaptation to the host. We found IncFII_1_pKP91 and IncFIB(K)_1_Kpn3, which have been previously reported in *K. pneumoniae* in Peru as carriers of resistance genes [39]. The IncFIB (K)_1_Kpn3 plasmid, found in two of our isolates, is known to be a megaplasmid that has facilitated the emergence and adaptation of MDR ESBL-producing strains of *Salmonella enterica* serovar Infantis [40]. Our results are consistent with a report where the IncFIB(K)_1_Kpn3 plasmid was linked to the *bla*_CTX-M_ gene, promoting the formation and spread of ST11-KL64 hv-CPKP strains [41].

Two *Klebsiella pneumoniae* isolates carrying the IncFIB(Mar) plasmid were identified, which plays a key role in the emergence and spread of hypervirulent and carbapenem-resistant strains (hv-CRKP) [42].

The resistome analysis revealed a broad distribution of resistance genes among the evaluated genomes, including those conferring resistance to beta-lactams and aminoglycosides, which were present in most of the isolates. The two ST307 isolates exhibited a similar multiresistance profile, but no carbapenem resistance genes were detected. Notably, the resistance gene *fosA6* was universally detected across all seven isolates. Given that 6/7 of the analysed infections in this cohort were urinary tract infections (UTIs). This finding carries direct, critical clinical implications. Regional epidemiological data from neighbouring Andean settings, such as the Bolivian Chaco, demonstrate that fosfomycin still retains low resistance rates (<10%) in community-onset UTIs, positioning it as an ideal empirical choice [43]. However, our detection of plasmid-borne *fosA6* in all of the evaluated clinical strains reveals a stark contrast. It underscores that genomic dissemination of fosfomycin resistance may already be silent but highly compromised in peripheral Peruvian hospital regions, heavily challenging current regional therapeutic guidelines for both uncomplicated and complicated UTIs.

The concordance between the phylogenetic clustering based on SNPs and the resistance profile indicates clonal similarity in certain isolates, while the variability observed in other profiles suggests horizontal transfer mediated by plasmids. Together, these results reflect the coexistence of clonal expansion and active genetic mobility within this small cohort. Due to the limited number of samples, these results represent an exploratory approach; nonetheless, they offer valuable insights into the evolutionary dynamics of these strains.

The virulome analysis revealed a diverse but distinct composition, with virulence scores ranging from 0 to 4 [31]. While core siderophores like enterobactin were universal, five out of seven isolates carried the acquired yersiniabactin (*ybt*) locus. Most importantly, isolate oph_54 (ST218) was identified as harbouring a convergent MDR-hvKP genomic profile strain, harbouring aerobactin (*iuc*) and salmochelin (*iro*) loci, a combination associated with high-risk hypervirulent lineages [44,45]. Globally, ST218 is recognised as a typical hypervirulent lineage (hvKP) that has recently demonstrated a high capacity to acquire mobile resistance elements, including transferable carbapenemase genes such as *bla*_NDM-1_, resulting in the critical emergence of MDR-hvKP convergent phenotypes [46]. In this study, the presence of *ybt* in isolates oph_20, oph_35, oph_43_r, and oph_53 suggests a significant evolutionary advantage, as this siderophore is not only essential for iron acquisition in iron-poor environments, such as the human urinary or respiratory tracts, but it also plays a critical role in evading host nutritional immunity by sequestering iron away from host proteins [47]. A critical finding in this study was the genomic profile of isolate oph_54, which reached a virulence score of 4. According to the Kleborate scoring system [], this indicates the presence of both aerobactin (*iuc*) and salmochelin (*iro*) loci. This co-occurrence of an MDR phenotype with high-risk virulence factors in a single isolate represents a concerning genomic convergence (MDR-hvKP) that poses a significant therapeutic challenge [48].

The genetic structure observed in the analysed isolates is consistent with the regional epidemiology of *K. pneumoniae* in Latin America, where the expansion of multidrug-resistant clones has been widely documented in hospital settings. Specifically, various studies in countries such as Brazil, Colombia, Mexico, and Peru have reported a growing prevalence of extended-spectrum beta-lactamase (ESBL)-producing strains, especially those carrying CTX-M family genes, which have become the main mechanism of resistance to third-generation cephalosporins in the region [49].

In summary, the presence of these clones in the analysed isolates suggests that the clinical settings evaluated are subject to complex genetic dynamics involving the circulation of *K. pneumoniae* lineages with a high potential for dissemination.

From a clinical perspective, these preliminary results highlight the urgent need for enhanced surveillance and infection prevention protocols. Genomic surveillance is essential for real-time monitoring of resistance and virulence evolution.

Study Limitations: This study has some limitations that should be acknowledged. First, the sample size is descriptive and exploratory due to the logistical constraints of performing WGS in peripheral regions of Peru. Second, all samples were handled strictly as anonymised residual isolates; therefore, detailed clinical records or patient outcomes could not be retrieved. Third, due to the lack of phenotypic virulence assays, the convergent strain oph_54 is characterised strictly at the genomic level as possessing an MDR-hvKP profile rather than assuming a definitive hypervirulent phenotype. Finally, sequencing was performed exclusively using Oxford Nanopore long-read technology without hybrid short-read polishing, which remains a technical limitation for specific single-nucleotide variations.

## 5. Conclusions

This study reports the presence of high-risk clones of *K. pneumoniae* circulating within the studied Peruvian hospitals, including ST307, ST11 and ST15, with ST307 being the most frequent among the analysed isolates, associated with the KL102 capsular type and the O1/O2v2 antigen. A significant finding was the identification of an MDR-hvKP genomic profile isolate (ST218, KL57) from the Loreto region, which was classified as a multidrug-resistant hypervirulent strain at the genomic level. These findings underscore the convergence of antimicrobial resistance and hypervirulence, posing a significant public health threat that warrants close monitoring.

The detection of a high plasmid burden and diverse resistance profiles suggests that clonal expansion and plasmid-mediated horizontal gene transfer both contribute to the genetic mobility of MDR *K. pneumoniae* in these hospital environments.

Overall, these findings emphasise the importance of genomic surveillance as a vital tool for understanding the evolutionary dynamics of *K. pneumoniae*, identifying high-risk clones and monitoring the emergence of strains that combine antimicrobial resistance and enhanced virulence. Even as an exploratory pilot, the implementation of WGS-based surveillance programmes could be highly valuable to support infection control strategies and inform antimicrobial stewardship policies in Peruvian healthcare settings.

## Figures and Tables

**Table 1:** Clinical characterisation of isolates obtained from patients.

Isolate ID	Region/Hospital	Gender	Age (Years)	Year	Clinical Source	Hospital Department
oph_14	Moquegua/Hospital Regional de Moquegua	F	76	2024	Urine	Internal Medicine
oph_20	Metropolitan Lima/Hospital de Huaycán	F	51	2025	Urine	Gynecology-Obstetrics
oph_35	Ucayali/Hospital Regional de Pucallpa	F	17	2025	Urine	Emergency- Gynecology
oph_43_r	Ucayali/Hospital Regional de Pucallpa	M	60	2024	Urine	Urology
oph_53	Loreto/Hospital Regional de Loreto	M	10	2025	Blood	ICU
oph_54	Loreto/Hospital Regional de Loreto	M	42	2025	Urine	ICU– Surgery
oph_85	Ucayali/Hospital Regional de Pucallpa	M	55	2025	Urine	Internal Medicine

Abbreviations: Isolate ID, clinical isolate identifier; oph, One Peru Human; ICU, Intensive care unit. Age expressed in years.

**Table 2:** Plasmids predicted from WGS contigs of *K. pneumoniae* MDR isolates.

Isolate ID	Replicon Type (PlasmidFinder)	Plasmid ID (MOB-Suite)	Replicon Family (MOB-Suite)	Size (kb)	Mobility	Resistance Genes (ABRICate)
oph_14	IncFIB(K)_1_Kpn3, IncFII_1_pKP91, Col440I_1	AA274	IncFIB, IncFII	228.9	Conjugative	*bla*_CTX-M-27_, *bla*_DHA-1_, *floR*, *aac(6′)-Ib-cr*, *arr-3*, *dfrA27*, *aadA16*, *aph(3′)-Ia*, *mph(A)*, *qnrB4*, *sul1*, *tet(D)*
		AC288	Unclassified	17.0	Non-mobilizable	*bla*_DHA-1_, *qnrB4*, *sul1*
oph_20	IncFII_1_pKP91, IncFIB(K)_1_Kpn3, ColRNAI_1	AA277	IncFIB, IncFII	251.0	Conjugative	*bla*_CTX-M-15_, *bla*_TEM-1B_, *bla*_OXA-1_, *aac(3)-IIa*, *aac(6′)-Ib-cr*, *aph(3″)-Ib*, *aph(6)-Id*, *dfrA14*, *qnrB1*, *sul2*, *tet(A)*
oph_35	IncHI1B_1_pNDM-MAR, IncFIB(Mar)_1_pNDM-Mar, IncFIB(K)_1_Kpn3, IncR_1, ColRNAI_1	AA405	IncFIB, IncFIB, IncHI1B	419.7	Conjugative	*bla*_CTX-M-15_, *aph(3′)-Ia*, *mph(A)*, *aadA2*, *dfrA12*, *aph(3″)-Ib*, *aph(6)-Id*, *qnrS1*, *sul1*, *sul2*
		AA277	Unclassified	144.5	Non-mobilizable	*bla*_CTX-M-15_, *bla*_TEM-1B_, *bla*_OXA-1_, *aac(6′)-Ib-cr*, *aac(3)-IIa, dfrA14, aph(6)-Id, aph(3″)-Ib, qnrB1, sul2, tet(A)*
		AC978	IncFIB	95.9	Conjugative	*bla*_OXA-1_, *dfrA14*, *aac(3)-IIa*, *aac(6′)-Ib-cr*, *tet(A)*
		AA117	ColRNAI	14.6	Mobilizable	None
oph_43_r	IncFIB(K)_1_Kpn3, IncHI1B_1_pNDM-MAR, IncFIB(Mar)_1_pNDM-Mar, ColRNAI_1	AA405	IncFIB, IncHI1B	264.8	Conjugative	*bla*_CTX-M-15_, *bla*_OXA-1_, *aac(3)-IIa*, *aac(6′)-Ib-cr*, *dfrA12*, *dfrA14*, *aph(3″)-Ib*, *aph(6)-Id*, *aph(3′)-Ia*, *mph(A)*, *aadA2*, *qnrS1*, *sul2*, *tet(A)*
		AA277	IncFIB	134.1	Non-mobilizable	*bla*_CTX-M-15_, *bla*_TEM-1B_, *aph(3″)-Ib*, *aph(6)-Id*, *qnrS1*, *sul2*
oph_53	IncFII_1_pKP91, IncFIB(K)_1_Kpn3	AA276	IncFIB, IncFII	194.9	Conjugative	*bla*_CTX-M-15_, *bla*_OXA-1_, *bla*_TEM-1B_, *aac(6′)-Ib-cr*, *dfrA14*, *tet(A)*, *aph(6)-Id*, *aph(3″)-Ib*, *qnrS1*, *sul2*
oph_54	IncFIB(K)_1_Kpn3, IncFII(pHN7A8)_1_pHN7A8, ColRNAI_1	AA406	IncFIB	216.7	Non-mobilizable	None
		AA327	IncFIA, IncFII	100.3	Conjugative	*bla*_CTX-M-55_, *bla*_TEM-141_, *ant(3″)-Ia*, *tet(A)*, *aph(6)-Id*, *aph(3″)-Ib*, *lnu(F)*, *sul2*
oph_85	IncX1_1, ColE10_1, IncN_1, Col440I_1	AA276	IncFIB, IncFII	149.1	Conjugative	*bla*_CTX-M-15_
		AA452	Unclassified	79.1	Non-mobilizable	*bla*_OXA-1_, *aac(3)-IIa*, *aac(6′)-Ib-cr*, *dfrA14*
		AA551	IncN, IncX1	48.1	Conjugative	None

Abbreviations: kb, kilobases. Plasmid replicons were screened using PlasmidFinder, while complete physical scaffolds, total size, and horizontal mobility traits were reconstructed via MOB-suite.

**Table 3:** Intra-isolate resistome diversity and antimicrobial gene distribution profiles based on Simpson’s Index of Diversity (1 − *D*).

Isolate ID	Sequence Type (ST)	Total AMR Genes (*N*)	Antimicrobial Class Distribution (*n*)	Simpson’s Index of Diversity (1 − *D*)
oph_14	ST11	5	Aminoglycosides (1), Beta-lactams (1), Quinolones (1), Trimethoprim (1), Fosfomycin (1)	1.000
oph_54	ST218	11	Aminoglycosides (3), Beta-lactams (2), Quinolones (1), Sulfonamides (2), Trimethoprim (1), Fosfomycin (1), Tetracyclines (1)	0.909
oph_35	ST307	13	Aminoglycosides (3), Beta-lactams (3), Quinolones (2), Sulfonamides (1), Trimethoprim (2), Fosfomycin (1), Tetracyclines (1)	0.897
oph_43_r	ST307-1LV	14	Aminoglycosides (4), Beta-lactams (3), Quinolones (2), Sulfonamides (1), Trimethoprim (2), Fosfomycin (1), Tetracyclines (1)	0.879
oph_85	ST15	7	Aminoglycosides (2), Beta-lactams (2), Sulfonamides (2), Fosfomycin (1)	0.857
oph_53	ST45	12	Aminoglycosides (4), Beta-lactams (3), Sulfonamides (2), Trimethoprim (1), Fosfomycin (1), Tetracyclines (1)	0.848
oph_20	ST307	12	Aminoglycosides (4), Beta-lactams (3), Sulfonamides (2), Trimethoprim (1), Fosfomycin (1), Tetracyclines (1)	0.848

Note: Isolates are ranked in descending order of resistome diversity. *N*, total number of antimicrobial resistance (AMR) genes; *n*, number of genes per specific antimicrobial class. Simpson’s Index of Diversity (1 − *D*) was calculated using the finite community framework; values closer to 1.000 indicate a highly heterogeneous and balanced distribution of resistance mechanisms across different drug families.

## Data Availability

All data regarding the results of this research are available in GenBank (BioProject PRJNA1458278).

## Supplementary Materials

The following supporting information can be downloaded at: https://www.mdpi.com/article/10.3390/microorganisms14071573/s1, Table S1: Detailed phenotypic antimicrobial susceptibility testing (AST) profiles of the *Klebsiella* clinical isolates; Table S2: Quality control metrics of sequencing reads and genome assemblies for *Klebsiella pneumoniae* isolates.

## Abbreviations

The following abbreviations are used in this manuscript:

MDR	Multidrug-resistant
WGS	Whole-Genome Sequencing
