# Supplementary material for: Exploratory Genomic Characterisation of the Resistome, Virulome and Molecular Epidemiology of Multidrug-Resistant Klebsiella pneumoniae Clinical Isolates from Four Peruvian Regions
Source: Microorganisms. 2026 Jul 19;14(7):1573. doi: 10.3390/microorganisms14071573 (PMC13413516; doi:10.3390/microorganisms14071573)
Supplement: Supplementary file 1 [file microorganisms-14-01573-s001.zip › microorganisms-4358102-supplementary.pdf]

Supplementary Table S1. Detailed phenotypic antimicrobial susceptibility testing (AST) profiles of the *Klebsiella* clinical isolates.

Categories	Carbapenems		Extended-spectrum cephalosporins (3rd and 4th generation)			β-lactamase inhibitors	Antipseudomonal penicillins + β-	Monobactams	Aminoglycosides		Fluoroquinolones	Non-fluorinated quinolones	Folate pathway inhibitors	Tetracyclines	Nitrofuran derivatives	Polymyxins	Non-Susceptible Categories	MDR*
Isolate ID	MEM	ERT	CAZ	CTX	CPM	AMC	PIP/ TAZ	ATM	AMI	GEN	CIP	NAL	SXT	MNO	NIT	COL	(Count ≥ 3)	Yes/No
oph 14	I	I	R	R	R	R	I	R	R	—	R	R	R	R	—	—	10/12	Yes
oph 20	S	—	R	R	R	—	R	—	—	—	R	R	—	—	—	—	4/12	Yes
oph 35	R	R	R	R	R	R	R	R	R	—	R	R	—	R	—	—	9/12	Yes
oph 43 r	S	—	R	R	R	R	R	R	R	—	R	R	R	R	—	—	9/12	Yes
oph 53	S	S	S	R	R	I	S	R	S	R	R	R	R	—	S	S	7/12	Yes
oph 54	S	S	S	R	S	S	—	S	S	I	R	R	R	—	S	S	4/12	Yes
oph 85	S	S	R	R	R	R	R	—	—	—	R	R	—	R	—	—	6/12	Yes

Abbreviations: MEM, meropenem; ERT, ertapenem; CAZ, ceftazidime; CTX, cefotaxime; CPM, cefepime; AMC, amoxicillin/clavulanic acid; PIP/TAZ, piperacillin/tazobactam; ATM, aztreonam; AMI, amikacin; GEN, gentamicin; CIP, ciprofloxacin; NAL, nalidixic acid; SXT, trimethoprim/sulfamethoxazole; MNO, minocycline; NIT, nitrofurantoin; COL, colistin. Interpretive categories: S, susceptible; I, intermediate; R, resistant; —, not determined (due to variations in regional hospital testing availability). Susceptibility breakpoints were interpreted according to the Clinical and Laboratory Standards Institute (CLSI, 2025) guidelines. *MDR status: Evaluated according to Magiorakos et al. (2012) criteria, defined as acquired non-susceptibility to ≥ 1 agent in ≥ 3 antimicrobial categories.

Supplementary Table S2. Quality control metrics of sequencing reads and genome assemblies for *Klebsiella pneumoniae* isolates.

Genome assemblies ranged from 5.6 to 5.9 Mb in size, with GC contents between 56% and 57%, consistent with the expected range for *K. pneumoniae*. All assemblies achieved 100% completeness and less than 3% contamination (CheckM), ensuring high-quality genomic data (Table S2). Notably, high contiguity was observed across all isolates, with N50 values exceeding 5.2 Mb, indicating a near-complete reconstruction of the bacterial chromosomes using Nanopore sequencing. Although slight variability in the number of contigs was observed in isolates such as oph_35 and oph_43_r, this did not compromise the identification of the full resistome and virulome, as the high N50 values ensured the integrity of large genomic islands and plasmids.

Isolate ID	Mean Quality (Phred Q)	Read N50 (kb)	Estimated Coverage (X)	Genome size (Mb)	GC (%)	Contigs	N50 (Mb)	Completeness (%)	Contamination (%)
oph_14	15.29	19.61	86.0x	5.64	57.00	5	5.36	100.0	0.29
oph_20	15.06	15.85	79.3x	5.80	56.99	4	5.47	100.0	1.80
oph_35	14.41	6.96	109.4x	6.56	56.04	40	3.49	100.0	2.18
oph_43_r	15.15	7.58	47.0x	5.86	56.70	17	5.37	100.0	0.63
oph_53	14.81	7.48	26.7x	5.44	57.00	2	5.24	100.0	0.10
oph_54	15.52	9.46	45.7x	5.60	57.13	4	5.27	100.0	0.22
oph_85	14.14	11.45	92.4x	5.70	56.84	8	5.35	100.0	0.16

Note: Phred Q, Phred quality score derived from Dorado basecalling; Read N50, the sequence length defining that 50% of total raw bases are in reads of that length or longer (in kilobases, kb); Estimated Coverage, genomic depth calculated from raw reads; Genome Size, total length of the assembled genome (in megabases, Mb); GC Content, percentage of guanine and cytosine bases; Contigs, number of contiguous sequences in the final assembly; Assembly N50, N50 metric calculated for the final assembled contigs (in megabases, Mb); Completeness and Contamination, checkM/CheckM2 genome quality estimates. All isolates achieved high-quality status (>99% completeness, <3% contamination).
